# Micronutrients in Support to The Carbon Cycle Activate Antioxidant Defences and Reduce Sperm DNA Damage in Infertile Men Attending Assisted Reproductive Technology Programs: Clinical Trial Study

**DOI:** 10.22074/ijfs.2020.6084

**Published:** 2020-02-25

**Authors:** Farzaneh Bassiri, Marziyeh Tavalaee, Maurizio Dattilio, Mohammad Hossein Nasr-Esfahani

**Affiliations:** 1 Department of Reproductive Biotechnology, Reproductive Biomedicine Research Center, Royan Institute for Biotechnology, ACECR, Isfahan, Iran; 2 R&D Department, Parthenogen, Piazza Indipendenza 11, Lugano 6900, Switzerland; 3 Isfahan Fertility and Infertility Centre, Isfahan, Iran

**Keywords:** Antioxidant, DNA Methylation, Male Infertility, Micronutrients, Sperm

## Abstract

**Background:**

Micronutrients in support to the carbon cycle were shown to reduce sperm DNA damage both in animal models and infertile men. Besides supporting DNA methylation, their positive effect may be mediated by an improved performance of the endogenous antioxidant system but this has not yet been proven in clinical settings. The present study aimed at evaluating the effects of micronutrient supplementation in infertile male partners of assisted reproductive technology (ART) resistant couples.

**Materials and Methods:**

In this experimental clinical trial study, infertile male partners of couples resistant to at least one ART cycle, with a sperm fragmentation rate >20% (TUNEL), underwent a 4-month oral supplementation with micronutrients in support to the carbon cycle including folates, B vitamins, zinc and cysteines. Semen, sperm DNA fragmentation (TUNEL), nuclear maturation (CMA3 and blue aniline staining) and lipid peroxidation (BODIPY) were assessed before and after treatment. The couples were followed-up to record clinical outcomes.

**Results:**

Forty-three patients completed the program but full data of pre- and post-treatment were available only for 25 patients. The treatment did not modify sperm concentration or motility but improved morphology. Nuclear maturation, DNA fragmentation and lipid peroxidation significantly improved after the treatment. Overall, 10 clinical pregnancies (23.3%) and 4 live births (9.3%) were recorded during the follow-up following expectant management (25 couples) or a new intracytoplasmic sperm injection (ICSI) cycle (18 couples).

**Conclusion:**

The micronutrients appeared to induce both DNA methylation, resulting in improved sperm nuclear matu- ration, and antioxidant defences, resulting in less DNA fragmentation and lipid peroxidation. The clinical outcomes were aligned with a possible positive effect on reproductive function. Micronutrients could be regarded as an alternative to antioxidants in correcting oxidative damage in infertile men; however, to confirm such findings, further clinical investigations are warranted (Registration number: IRCT201510207223N6).

## Introduction

Oxidative stress has been recognized as a main cause of male subfertility with sperm DNA damage affecting fertilization rates, embryo quality and pregnancy rates within assisted reproductive technology (ART) cycles (1). This has triggered efforts to improve the male fertility, and possibly the ART outcomes, by administering oral antioxidants. The latest available Cochrane review on male infertility (2) concluded that although the quality of evidence is weak, oral antioxidants significantly improve the chances of live birth for couples attending fertility clinics, which confirms the primary role of oxidative imbalance in male infertility. The evidence is so far weaker than expected, mostly due to the low quality of available studies. It is very difficult to establish the amount of antioxidants needed in the single subject and too strong supports may result in excess sperm nuclear decondensation and worsening of male reproductive potential (3), which has been defined as "reductive stress"(4). The available products often contain high doses of antioxidants and multiple exposures, including food fortification, may easily lead to excess of antioxidants with possible negative effects (5).

**Fig 1:**
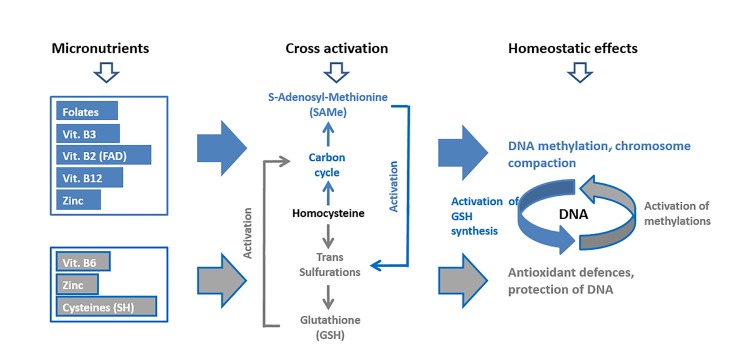
Concept model of micronutrients administration in support to DNA methylation (blue pathway) and antioxidant defences (grey pathway). Micronutrients: small amounts of folates, vitamins B2, B3 and B12 and zinc are needed daily to feed the carbon cycle and the methylations (SAMe). The same applies to vitamin B6, zinc and cysteine to feed glutathione (GSH) synthesis. Cross activation: SAMe acts on the enzyme CBS to increase GSH synthesis. Reducing power from GSH synthesis in turn activates the carbon cycle. Homeostatic effects: DNA methylation and antioxidant defences synergize in keeping a healthy DNA status.

Under physiologic conditions, the oxy-redox balance, besides leveraging on the assumption of reducing substances with diet, is largely based on the endogenous production of reducing power in the form of glutathione (GSH), a reducing tripeptide with an activated sulfhydryl group (SH) able to donate reducing equivalents and reactivate most of the physiologic antioxidants (6). GSH de novo biosynthesis occurs within the one carbon cycle, responsible for DNA methylation and epigenetics, by transulfuration of its end-product, homocysteine, to cysteine. Cysteine is then complexed with glutamate and glycine to form GSH. The ability of the carbon cycle to support DNA methylation is well-known to be regulated by the availability of dietary micronutrients (7) and this may also be true for the induction of GSH synthesis and antioxidant defences. Indeed, the functions of DNA methylation and antioxidant power generation are cross regulated and may respond to the same micronutrients within a homeostatic unit named "methoxistasis"(8). Methyl donors (e.g. folates), vitamins B2, B3 and B12 and zinc are essential to activate the methylations, which in turn result in activation of GSH synthesis. Vitamin B6, zinc and extra cysteines directly feed GSH synthesis and thus, facilitate the methylations. The concept of micronutrients administration in support to DNA methylation and GSH synthesis, is depicted in Figure 1.

The above substances administered to male partners of couples resistant to ART due to a male factor, resulted, differently from what seen with strong oral antioxidants (9), in a significant correction of both sperm DNA fragmentation and nuclear decondensation suggesting that a boost to the natural antioxidant defences may be effective and devoid of rebound effects (10). Many of the treated patients achieved a pregnancy, either spontaneously or following a new ART cycle, and the pregnancies were strongly correlated with the improvement of sperm nuclear condensation. This was expected due to the known ability of these micronutrients to support DNA methylation (7) and the ability of DNA methylation to trigger chromosome compaction (11). The nuclear compaction could also improve the resistance of DNA to oxidative attacks resulting in less DNA fragmentation. Thus, the induction of the endogenous antioxidant system has not been definitively proven. We tested the same micronutrients in a model of varicocele induction in rats (12) and found that the improvements of sperm DNA damage were paralleled by a sharp reduction of lipid peroxidation, which vouches for a strong induction of antioxidant activity. The present study intended to test the same combination of micronutrients in ART-resistant infertile men and confirm that its effect is linked to a true antioxidant induction as previously seen in the animal model.

## Materials and Methods

### Patients and study design

This experimental clinical trial study was approved by the Ethical Committee of Royan Institute (IR.ACECR. Royan.REC.1394.9) and carried out between April 2015 and April 2018 at the Isfahan Fertility and Infertility Center, Iran.

We included couples with male factor infertility, at least 1 failed ART cycle [either intra uterine insemination (IUI), *in vitro* fertilization (IVF) or intracytoplasmic sperm injection (ICSI)] and a male partner with high rate of sperm DNA fragmentation (>20% as measured by TUNEL) with no female factor. Female partners were defined as normal based on regular ovarian cycles, normal hormonal profile and no findings found in the hysterosalpingogram. All subjects were informed about the details of the study and a consent form was signed by both partners.

The male partners of the enrolled couples were prescribed with a 4-month treatment with a nutritional supplement containing micronutrients in support to the carbon cycle: folic acid (800 μg), vitamins B2 (2.8 mg), B3 (32 mg), B6 (2.8 mg) and B12 (5 μg), zinc (25 mg) and N-acetyl cysteine (500 mg) per day. Sperm quality was assessed at baseline and the end of the treatment.

The couples were followed-up for 3 to 10 months from treatment termination and pregnancies, either spontaneous or following a new ICSI cycle, were recorded.

### Sperm quality testing

The semen sample was collected by masturbation after 3-7 days of abstinence before and after the supplementation. Sperm parameters were assessed according to the WHO (2010) criteria and motility was assessed by CASA (CASA, Video Test, ltd: version Sperm 2.1© 1990-2004, Russia). DNA fragmentation was assessed by TUNEL (Apoptosis Detection System Fluorescein, Promega, Germany) as previously reported (13). Lipid peroxidation was assessed using BODYPI as previously shown (14). Chromatin condensation and maturation were assessed by CMA3 and aniline blue staining, respectively (15, 16).

### Sperm preparation and intracytoplasmic sperm injection procedure

In case of couples undergoing a new ART cycle posttreatment, ovulation induction in the female partner was achieved by administration of recombinant follicle stimulating hormone (FSH, Sinal F, SinaGene, Iran) and human menopausal gonadotropin (hMG, Menogon, Ferring, Germany) after pituitary suppression by a gonadotropin release hormone (GnRH) antagonist (Cetrotide, Merk-Serono, USA). Follicular maturation was monitored by trans-vaginal ultrasounds and final ovulation was induced by 10000 IU human corionic gonadotropin (hCG, Choragon, Ferring, Germany). Oocytes were collected using ultrasound-guided trans vaginal aspiration and cumulus and coronal cells were removed to evaluate oocyte maturity. MII oocytes were selected for the following ICSI procedure.

Partner’s sperm was prepared for ICSI by density gradient centrifugation (13). Morphologically normal and motile sperm were selected under 200-400X magnification and injected into the MII oocytes. Inseminated oocytes and embryos were cultured at 37°C in 6% CO2, 6% O2 under humidified conditions. ICSI and the following embryo culture, were performed *in Vitro* life culture media (G-V series, Vitrolife, Sweden).

Fertilization, cleavage, implantation, clinical pregnancy and abortion rate were defined and assessed based on the terminology of the international committee for monitoring assisted reproductive technologies (ICMART) (17). Embryo quality was assessed on day 3 according to f Giorgetti et al. (18) and Terriou et al. (19). Embryos with 6-8 cells, equal blastomere size and less than 25% fragmentation, were rated as "top quality" and fresh transferred or vitrified for later use. The fertilization, cleavage and top quality embryo rates were compared to those achieved by the same couples in their previous ICSI cycle.

### Statistical analysis

Statistical Package for the Social Sciences software (SPSS 18, Chicago, IL, USA) was used for data analysis. Data are expressed as mean ± error of the mean (SEM) and differences were considered significant at P<0.05. Comparison of sperm parameters, chromatin status, and lipid peroxidation, before and after treatment and clinical outcomes (fertilization, cleavage rate, top quality embryos) between current cycle and previous cycle was performed by Student’s t test. For comparison of pregnancy rate, Chi-square was used.

## Results

In total, 51 patients were enrolled and 8 of them quit for personal reasons. Data on semen analysis preand post-treatment were available for all 43 patients completing the study whereas some data concerning TUNEL (n=36), BODIPY, CMA3 and blue aniline (n=25) methods, were missing.

### Effect of micronutrients on sperm quality

**Fig 2:**
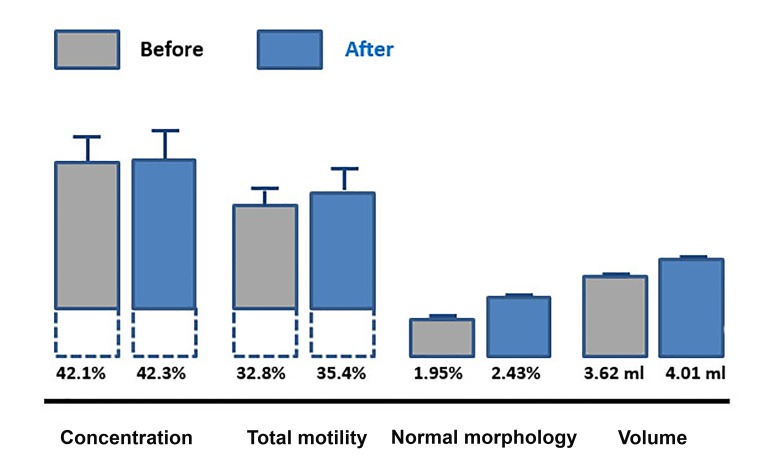
Sperm concentration (P=0.8), total motility (P=0.4), normal morphology (P=0.001), and volume (P=0.06) before and after a 4 month exposure to micronutrients, mean values ± SE.

**Fig 3:**
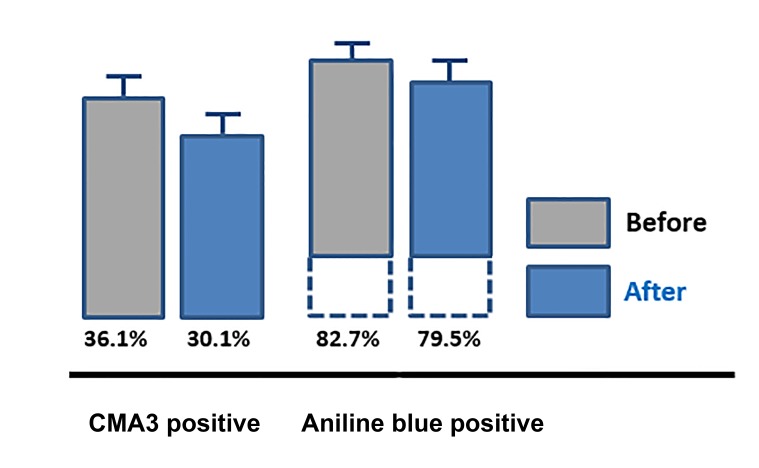
Sperm protamine deficiency (P<0.05) and nuclear maturation (P<0.001) before and after a 4 month exposure to micronutrients, mean values ± SE (n=24). CMA3 reports on sperm protamine deficiency, Aniline blue reports on sperm nuclear maturation.

**Fig 4:**
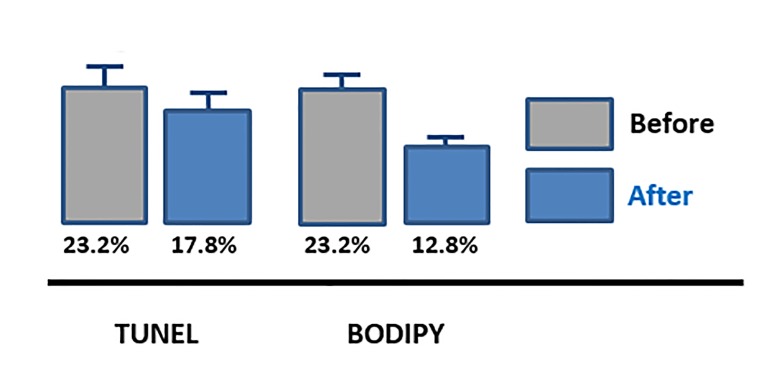
Sperm DNA fragmentation (P=0.001) and lipid peroxidation (P<0.001) before and after a 4 month exposure to micronutrients, mean values ± SE (n=24). TUNEL reports on sperm DNA fragmentation, BODIPY reports on lipid peroxidation.

The supplementation with micronutrients had no effect on sperm concentration, motility and volume but significantly improved sperm morphology (n=43, P=0.001, Fig .2). The treatment was of benefit with respect to sperm nuclear maturation with a significant reduction of the rate of sperms stained by CMA3 (n=24, P<0.05) and aniline blue (n=24, P<0.001, Fig .3). Micronutrients also improved oxidative damageinduced sperm DNA fragmentation measured by TUNEL (n=36, P=0.001) and lipoperoxidation measured by BODIPY (n=24, P<0.001), that significantly improved after treatment (Fig .4).

**Fig 5:**
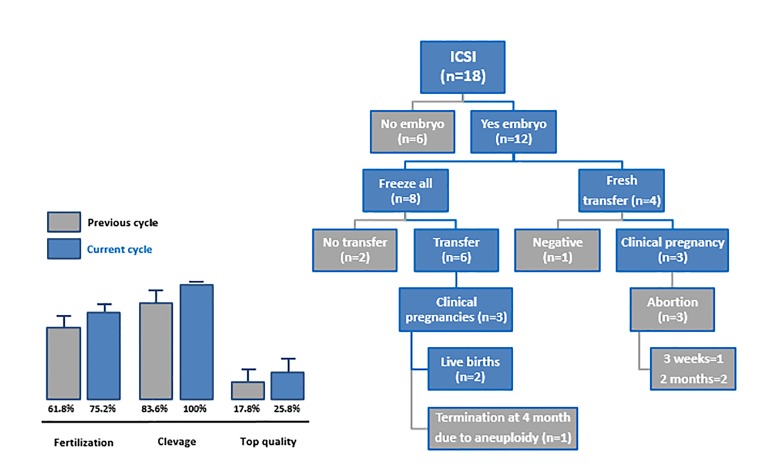
Outcomes from the ART cycles and couples disposition. Fertilization, cleavage (P<0.05) and top quality embryo rates were compared to those achieved by the same couples in their previous ICSI cycle. ART; Assisted reproductive technology and ICSI; Intracytoplasmic sperm injection

### Clinical outcomes

Out of the 43 couples whose male partner completed the treatment, 25 couples did not undergo a new ART cycle and opted for an expectant management strategy. Four of these couples, achieved a spontaneous pregnancy during the study follow-up period resulting in 2 deliveries and 2 spontaneous miscarriages.

The remaining 18 couples underwent a new ICSI cycle but only 12 of them had embryos suitable for transfer or vitrification. Four of them underwent a fresh transfer cycle resulting in 3 clinical pregnancies and 3 abortions at either 3 weeks (n=1) or 2 months (n=2). Only 6 of 8 couples with vitrified embryos, underwent a thawed embryo transfer during the study period resulting in 3 clinical pregnancies with 2 deliveries and one early termination due to aneuploidy. The embryo cleavage rate was significantly higher (P<0.05) compared to the one in the previous cycle in the same couples whereas the improvement of fertilization rate and top quality embryo rate was not significant. The couples’ disposition and outcomes after a new ICSI cycle are summarized in Figure 5. Overall, treatment of 43 male partners of ARTresistant couples with micronutrients in support to the carbon cycle, resulted in 10 clinical pregnancies (23.3%) and 4 live births (9.3%).

## Discussion

This was a small size explorative study aimed at testing the hypothesis that a nutritional intervention using micronutrients in support to the carbon cycle, is of benefit to infertile men attending ART programs and that it is able to induce both improved DNA methylation and resumption of the endogenous antioxidant activity.

The administration of micronutrients did not modify sperm count or motility but improved sperm morphology, which can be assumed as a possible effect on the processes of DNA methylation that contribute to the epigenetic programming of the cell phenotype. The parallel improvement of sperm nuclear maturation shown by CMA3 and blue aniline staining, also points to a possible effect on DNA methylation. Indeed, DNA and histone methylation plays a pivotal role in sperm nuclear maturation and is also involved in the process of protamination (20). The induction of DNA methylation is explained by the ability of the administered folates to feed homocysteine re-methylation to methionine. Methionine is adenylated to generate the universal methyl donor S-adenosylmethionine (SAMe) acting as a substrate for DNA N-methyltransferases. We also supplemented vitamin B12 that is needed to pass the methyl group from folates to homocysteine, vitamins B2 and B3 as cofactors for methylentetrahydrofolate reductase (MTHFR) to activate folates, and zinc that is as well necessary to MTHFR and methionine synthase for homocysteine remethylation.

Micronutrients supplementation achieved a significant reduction of sperm DNA fragmentation and lipid peroxidation. The improvement of DNA fragmentation was likely of clinical relevance because the average rate moved from 23.2 to 17.8% (i.e. it dropped below the critical threshold of 20% that is assumed as clinically relevant) (21). This may imply an improved performance of the endogenous antioxidant metabolism because the administered micronutrients did not include any direct antioxidant substances. However, the origin of sperm DNA fragmentation is controversial with several processes showing the ability to contribute. According to Muratori et al. (22), oxidative aggression does not behave as the primary trigger, rather it is a process of apoptosis, likely triggered by an impairment of chromatin maturation in the testis and oxidative stress during the transit in the male genital tract. Therefore, the positive effect of micronutrients on DNA fragmentation may be already explained by the protection from an improved chromatin packaging resulting from the induction of methylations. However, an improvement of the antioxidant defences may also be involved.

Micronutrients also achieved a significant drop in sperm lipid peroxidation to half of the baseline value. Lipid peroxidation is a process where oxidants attack lipids containing carbon-carbon double bond(s), especially polyunsaturated fatty acids (PUFAs), resulting in production of other radicals attacking other double bonds in a self-amplified process and is a clear-cut oxidative damage (23). The abundance of PUFAs in the sperm membrane is a reason for the particular sensitivity of sperms to lipid peroxidation if an oxidative imbalance occurs (24). GSH is produced as a reaction to oxidative stress by activation of the redox-sensitive transcription factor, nuclear factor erythroid-2-related factor 2 (Nrf2), that activates the enzyme γ-glutamyl cysteine ligase. This enzyme is further regulated by the availability of intracellular cysteines from both the diet and endogenous synthesis (25). Our micronutrients included soluble cysteine in the form of N-acetylcysteine and supported the endogenous cysteine synthesis by zinc and vitamin B6 acting as the necessary co-factor for cystathinonine β-synthase (CBS), the enzyme addressing homocysteine from the carbon cycle to cystathionine and then, cysteine synthesis. These micronutrients had therefore the ability to support antioxidant defences by facilitating GSH synthesis and the reduction of lipid peroxidation was likely a sign of antioxidant defences induction.

In addition, a synergy with other micronutrients is likely to apply. Abundance of SAMe from the carbon cycle can bind CBS resulting in an allosteric activation with a fivefold increase of enzyme activity (26). Thus, the administered micronutrients had the potential to induce a strong boost to intracellular cysteine availability by increasing both its nutritional intake and the endogenous synthesis for a better response of the GSH system to an increased oxidative load and this may account for the effect on lipid peroxidation. Worth to note, the synthesis of cysteine from homocysteine is the only source of the intracellular gasotransmitter H_2_S (27). H2S in turn promotes the activation of vitamin B12 (28) and plays as a main inducer of the carbon cycle designing a cross activated homeostatic unit.

Our study was too small in size to provide meaningful clinical findings; however, the outcomes were aligned with a possible positive effect of the treatment on the patients reproductive performance. Four out of 25 (16%) couples opting for an expectant management strategy, achieved a clinical pregnancy during the follow-up period. Interestingly, the male partners of these couples also showed a good response to the micronutrients of the sperm damage indexes (data not shown), which endorses a possible link between the positive pregnancy outcome and the treatment. Out of 18 couples opting for a new ART cycle, only 12 had viable embryos and only 10 of them underwent either fresh (n=4) or thawed (n=6) embryo transfer, which resulted in 6 clinical pregnancies. Again, the male partners of the couples achieving a pregnancy were good responders for sperm parameters. Altogether, these clinical outcomes indicate good chances that the treatment can be of help to male reproductive function at least in good responders. Reasons for lack of response may be bad treatment compliance, a negative genetic background and occurrence of other pathogenic mechanisms beside oxidative aggression, but our data do not allow to further speculate on this.

In summary, the oral administration of a combination of micronutrients including folates, B vitamins, zinc and cysteines to male partners of ART-resistant couples, showed the ability to reduce sperm DNA damage and improve sperm nuclear maturation and the clinical outcomes reflected a positive effect. These outcomes were related to the ability of the micronutrients to activate the one carbon cycle resulting in both stronger methylation ability and activation of the endogenous antioxidant defences. The recorded antioxidant effect, which was strong and likely of clinical value, was achieved without perturbations of the cell homeostasis as happen with oral antioxidants (9).

## Conclusion

The present study failed to address a series of relevant questions including the actual clinical gain that can be achieved by micronutrients, how to individuate potential good responders, dose and duration of the treatment and whether the add-on of oral antioxidants may further improve the effect or rather just derange the homeostatic regulations. All of these questions should be addressed by larger size clinical trials centred on the clinical outcomes. Meantime, micronutrients qualify as an alternative to antioxidants in correcting oxidative damage in infertile men.
